# Efficacy and Safety of Risperidone Microspheres for Injection (II) in Patients With Schizophrenia Spectrum Disorders: A Prospective, Single-Arm, Multicenter Real-World Study

**DOI:** 10.31083/AP39454

**Published:** 2026-04-22

**Authors:** Wei Li, Jinghui Tong, Xiaoxiao Gao, Hu Deng, Zezhi Li, Junhong Zhu, Jiahong Liu, Yanchi Zhang, Dengtang Liu, Xiaobin Zhang, Jianping Tang, Yuxiu Sui, Hezeng Yang, Yunshu Zhang, Chao Li, Xuhui Zhou, Baoping Xing, Yi Wang, Jiaqi Song, Rongzhen Zhang, Yang Shen, Yanying Song, Yanfang Zhou, Shujuan Pan, Wei Zheng, Yunlong Tan

**Affiliations:** ^1^Peking University HuiLongGuan Clinical Medical School, Beijing HuiLongGuan Hospital, 100096 Beijing, China; ^2^The Affiliated Brain Hospital of Guangzhou Medical University, Key Laboratory of Neurogenetics and Channelopathies of Guangdong Province and The Ministry of Education of China, Guangzhou Medical University, 510370 Guangzhou, Guangdong, China; ^3^The Affiliated Wuhan Mental Health Center of Tongji Medical College, Huazhong University of Science and Technology, 430030 Wuhan, Hubei, China; ^4^Zhejiang Provincial Clinical Research Center for Mental Health, The Affiliated Kangning Hospital of Wenzhou Medical University, 325007 Wenzhou, Zhejiang, China; ^5^Department of Psychology, The Sixth Hospital of Changchun, 130052 Changchun, Jilin, China; ^6^Shanghai Mental Health Center, School of Medicine, Shanghai Jiao Tong University, 200030 Shanghai, China; ^7^The Affiliated Guangji Hospital of Soochow University, 215137 Suzhou, Jiangsu, China; ^8^Affiliated Mental Health Center & Hangzhou Seventh People’ s Hospital, Zhejiang University School of Medicine, 310013 Hangzhou, Zhejiang, China; ^9^Department of Psychiatry, Nanjing Brain Hospital, 210029 Nanjing, Jiangsu, China; ^10^Department of Psychiatry, Shenzhen Kangning Hospital, 518118 Shenzhen, Guangdong, China; ^11^Hebei Key Laboratory of Major Mental and Behavioral Disorders, The Sixth People's Hospital of Hebei Province, 071000 Baoding, Hebei, China; ^12^Department of Clinical psychology, Xi'an Mental Health Center, 710061 Xi'an, Shaanxi, China; ^13^Department of Addiction Medicine, Brain Hospital of Hunan Province (The Second People's Hospital of Hunan Province), 410007 Changsha, Hunan, China; ^14^Zhejiang Mental Health Center, Tongde Hospital of Zhejiang Province, 310012 Hangzhou, Zhejiang, China; ^15^Tianjin Anding Hospital, Mental Health Center of Tianjin Medical University, 300222 Tianjin, China

**Keywords:** schizophrenia spectrum disorders, long-acting injectable antipsychotics, risperidone microspheres, real-world study, positive and negative syndrome scale

## Abstract

**Background:**

Long-acting injectable (LAI) antipsychotics improve adherence and promote long-term recovery in schizophrenia spectrum disorders (SSD). Risperidone Microspheres for Injection (II) (RMI-II), a novel LAI formulation, offers rapid symptom control with a 2-week dosing interval. However, real-world evidence regarding its efficacy and safety remains limited.

**Methods:**

A prospective, single-arm, multicenter study was conducted across 15 Chinese research centers. Eligible patients (n = 228) met DSM-5 criteria for SSD, had a Positive and Negative Syndrome Scale (PANSS) total score ≥70, and were followed for 12 weeks. Patients received RMI-II (25–50 mg/2 weeks) via intramuscular injection.

**Results:**

Among 228 patients (50.88% male, mean age [37.00 ± 12.82] years) receiving RMI-II, the PANSS total score decreased significantly by 14.02 (3.22, 24.85) at week 2 (*p* < 0.001) and 38.28 (19.02, 51.34) at week 12 (*p* < 0.001). At week 2, 37.1% patients achieved clinical response (defined as ≥20% reduction in PANSS total score), and this proportion increased to 83.7% by week 12. By the end of the 12-week treatment, the clinical remission rate (defined as scores ≤3 on PANSS items P1, P2, P3, N1, N4, N6, G5, and G9) was 68.9%. The Clinical Global Impression–Severity Scale score improved from a baseline moderate-to-severe level 5.0 (5.0, 6.0) to a mild-to-moderate level 3.0 (2.0, 4.0). Adverse events occurred in 15.35% of patients, with hyperprolactinemia (1.3%) and extrapyramidal symptoms (1.3%) being most common.

**Conclusions:**

RMI-II appeared to be effective and well-tolerated in reducing acute psychotic symptoms. These findings suggest it may represent an additional therapeutic option for SSD.

**Clinical Trial Registration:**

No: ChiCTR2200066865, 20 December 2022, https://www.chictr.org.cn/showproj.html?proj=183302.

## Main Points

1. In real-world study, Risperidone Microspheres for Injection (II) (RMI-II) demonstrated to be effective in the early stage in patients with schizophrenia spectrum disorders (SSD).

2. With the long-term use of RMI-II, patients experienced further remission and did not exhibit significant fluctuations by week 12.

3. RMI-II were associated with minor adverse events, with hyperprolactinemia and extrapyramidal symptoms being the most common.

4. RMI-II have shown efficacy in improving multidimensional psychotic symptoms during the acute phase of SSD, exhibiting a favorable safety and tolerability profile.

## 1. Introduction

Schizophrenia spectrum disorders (SSD), including schizophrenia, schizoaffective disorder, delusional disorder and so on, affect approximately 0.3%–0.7% of the global population, leading to significant disability and societal burden [1, 2, 3, 4]. Oral antipsychotics play a key role in the treatment of SSD, however nonadherence to medicine remains a major challenge, contributing to high relapse rates (60%–80% within 2 years) [5, 6], and even relating to treatment resistance [7]. Periods of exacerbated active symptoms leads to repeated hospitalizations, loss of productivity, incarceration, and mortality [8]. Long-acting injectable (LAI) antipsychotics address adherence issues by ensuring steady drug delivery, thereby stabilizing symptoms and reducing relapse risk [9, 10].

Risperidone, a second-generation antipsychotic [11], has been formulated into LAIs such as the first risperidone microsphere formulation (RM-I), which has been shown to be effective in maintaining symptom control, reducing risk of relapse, and delaying time to relapse in schizophrenia [12, 13]. However, RM-I requires biweekly administration after an initial oral overlap [14], and its delayed therapeutic onset (3–4 weeks) restricts its utility in acute settings [15]. Beyond RM-I, newer LAI risperidone formulations include Risperidone Microspheres for Injection (II) (RMI-II, intramuscular biweekly injection), risperidone *in situ* microparticles (ISM, intramuscular monthly injection), RBP-7000 (subcutaneous monthly) and TV-46000 (subcutaneous monthly/bimonthly) [16]. RMI-II (developed by Shandong Luye Pharmaceutical Co., Ltd.), a modified formulation utilizing poly (lactic-co-glycolic acid) (PLGA), achieves rapid plasma concentration without a lag phase, enabling immediate symptom control [17]. Studies reported earlier C_max_ achieved between days 14 and 17 for RMI-II and days 32 and 34 for RM-I at doses of 25 mg and 50 mg [17, 18]. The sustained release profile maintains effective plasma concentrations over 4–5 weeks. Notably, steady-state pharmacokinetics are approximated following the second injection, obviating the requirement for concomitant oral risperidone supplementation during initiation therapy [18]. On the other hand, the elimination of RMI-II was completed approximately 2 weeks earlier as compared to that for RM-I [19]. Overall, RMI-II was safe and well tolerated, with a faster onset and offset, and demonstrated bioequivalence at steady state compared to RM-I.

RMI-II received a priority review designation from the Center for Drug Evaluation (CDE) under the National Medical Products Administration (NMPA) in December 2019 and was approved for market launch by the NMPA on January 12, 2021. Additionally, RMI-II was approved for listing in the USA in January 2023 and is currently undergoing global registration processes. Despite its approval for the treatment of SSD and its promising pharmacokinetic profile, real-world data on RMI-II are limited. This study aimed to assess its efficacy, safety, and usage strategy across a diverse SSD population.

## 2. Methods

### 2.1 Study Design and Participants

This study was a prospective, single-arm, multicenter Real-World study (ChiCTR2200066865) aimed at evaluating the treatment strategy, efficacy, and safety of RMI-II in adult patients with SSD. The project was led by Beijing Huilongguan Hospital and involved a total of 15 research centers in its implementation. Study was approved by the independent ethics committees of the respective sites (2022-49-drug) and conducted in accordance with the Declaration of Helsinki [20], Good Clinical Practice, and applicable regulatory requirements. All patients provided written informed consent for participation in this study.

**Fig. 1.**
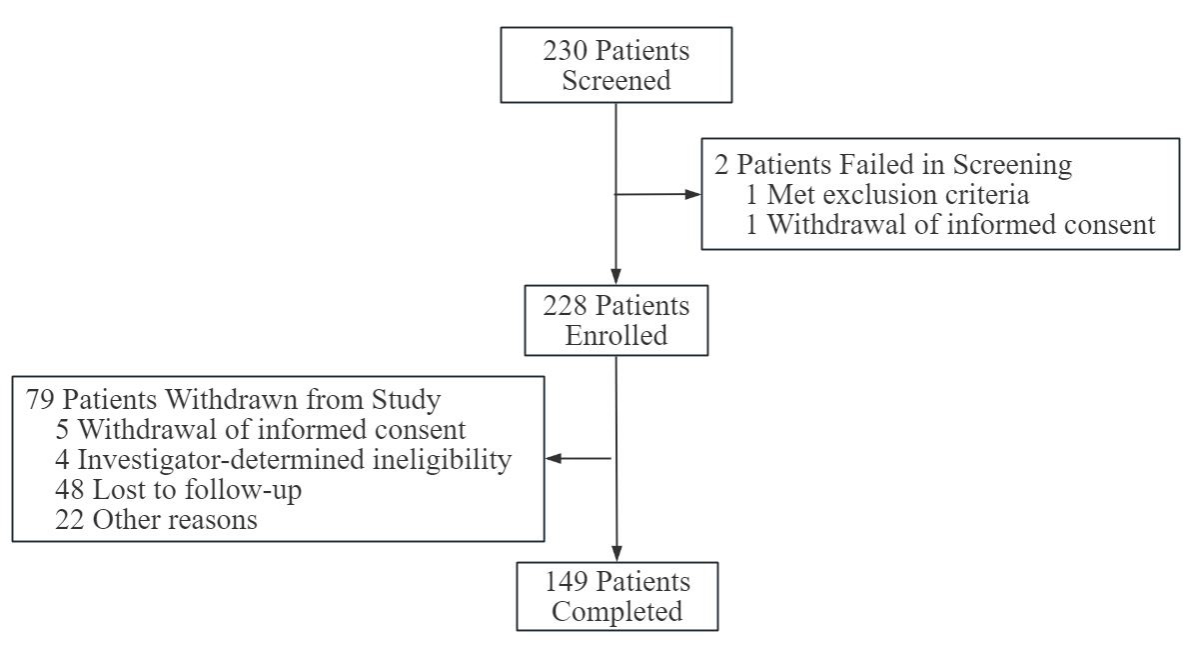
**Study participant flowchart**.

The study enrolled SSD patients from 15 Chinese psychiatric centers between February 2023 and June 2024 (Fig. 1). Eligible participants were adults (18–65 years) meeting DSM-5 criteria for SSD, with a Positive and Negative Syndrome Scale (PANSS) [21] total score ≥70 and at least one severe positive symptom (score ≥4 on P1 [Delusions], P2 [Conceptual Disorganization], P3 [Hallucinatory Behavior], P6 [Suspiciousness/persecution], or G9 [Unusual Thought Content]). Exclusion criteria included the patients: (1) with psychiatric diagnoses other than SSD; (2) with severe somatic diseases, intellectual disability, or abnormal laboratory parameters (Alanine Aminotransferase/Aspartate Aminotransferase [AST/ALT] ≥ 2 × upper limit of normal [ULN], Cr > 1.2 × ULN, QTc interval >450 ms in males or >470 ms in females); (3) with treatment-resistant schizophrenia; (4) with history or current presence of tardive dyskinesia (TD), neuroleptic malignant syndrome (NMS), or severe extrapyramidal adverse reactions; (5) with history of hypersensitivity or non-response to risperidone or paliperidone; (6) with substance abuse, and so on.

### 2.2 Intervention

Patients were administered RMI-II (Shandong Luye Pharmaceutical Co., Ltd., Yantai, Shandong, China, National Drug Approval Numbers: H20210001, H20210002, H20210003. Strengths: 25 mg, 37.5 mg, and 50 mg) at doses ranging from 25 to 50 mg via intramuscular (IM) injection into alternating gluteal muscles every two weeks for a period of 12 weeks. Dose adjustments were allowed based on individual tolerability and symptom response. (Patients with no prior exposure to risperidone or paliperidone began with oral risperidone 1 mg once daily for two consecutive days to evaluate tolerability; those stabilized on oral risperidone 1–2 mg/day started with 25 mg IM every two weeks, and patients on oral risperidone ≥3 mg/day initiated with 37.5 mg IM every two weeks).

### 2.3 Assessments 

The change from the baseline to each assessment visit at weeks 2, 4, 8, 12 in PANSS was established as primary efficacy indicator. The change of scores on the Clinical Global Impression–Severity (CGI-S) Scale [22] (with a score range of 1–7, where higher scores denote greater illness severity) was included as secondary efficacy indicator. For patients who withdrew from the study before the end of the treatment period, all end-of-treatment assessments were conducted at the early termination visit. To ensure consistent use of the assessment tools, all raters underwent training and certification, and the intraclass correlation coefficient (ICC) was maintained at ≥0.80. The safety indicators encompassed treatment-emergent adverse events (TEAEs), mental status examination, injection site evaluation, weight change, and clinical laboratory test.

### 2.4 Statistical Analysis 

The Full Analysis Set (FAS) comprised eligible cases and discontinued cases, except excluded cases. For missing primary efficacy endpoints, the intention-to-treat (ITT) analysis principle was applied, utilizing the last observation carried forward (LOCF). Missing values in comparability analyses and secondary efficacy endpoints were not imputed (data-carry-forward) and were analyzed based on actual data available in the FAS. The Safety Set (SS) consists of all participants who received at least one dose of treatment and had recorded safety data. Missing safety data were not imputed. This set included partially evaluable excluded cases (e.g., those exceeding age inclusion criteria) but excluded cases where prohibited medications precluded safety assessments. Adverse event incidence rates were calculated using the SS as the denominator. Data were analyzed using Statistical Analysis System (SAS, version 9.4, SAS Institute, Cary, NC, USA). Continuous variables were summarized as mean ± SD (normal distribution) or median (Q1, Q3) (non-normal distribution); while categorical variables as frequencies. In analyzing repeated measurement data with repeated measures analysis of variance (ANOVA), the data did not pass the normality test. Since the study primarily focused on comparing each assessment visit with the baseline, the Wilcoxon signed-rank test was utilized. Furthermore, the Bonferroni correction was applied to reduce the occurrence of Type I errors. Missing data were handled via LOCF for primary outcomes. All statistical tests were two-sided, with a significance threshold of *p* ≤ 0.05 or *p* ≤ 0.05/4 (Bonferroni correction).

## 3. Results

### 3.1 Demographics, Clinical Characteristics

A total of 228 SSD patients were enrolled in this real-world study, and a total of 149 patients completed the whole study, with a completion rate of 65.35% (Fig. 1). Among the 228 participants in FAS, the average age was (37.00 ± 12.82) years, 116 (50.88%) were male and 112 (49.12%) were female. The mean body mass index (BMI) was (24.34 ± 4.25) kg/m^2^, the mean duration of the disease was (10.99 ± 9.29) years, and the mean age of onset was (26.05 ± 9.22) years. 14.47% of patients have a family history of mental illness, and 76.75% of patients have taken basic medication. Baseline PANSS total score was 92.0 (79.0, 106.0), indicating severe psychotic symptoms (Table 1). A total of 151 patients in this study had concomitant medication (66.23%, Table 2), including oral antipsychotics, mood stabilizers, antidepressants, anxiolytics, sedative-hypnotics, antiextrapyramidal symptom medications, β-adrenergic antagonists, glucose-lowering agents, and laxatives. 79 patients withdrew from the study (23 at week 2, 30 at week 4, 69 at week 8, and 79 at week 12), with only one withdrawal due to poor disease control, none withdrew due to safety events, and the primary reason was loss to follow-up.

**Table 1.** **Baseline demographics and clinical characteristics**.

Characteristic	Patients (n = 228)^a^
Age^b^	37.00 ± 12.82
Sex, male/female	116/112
Baseline BMI (kg/m^2^)	24.34 ± 4.25
Age at first diagnosis of SSD, year	26.05 ± 9.22
Duration of illness, year	10.99 ± 9.29
Family history, positive/negative	33/195
PANSS total score	92.0 (79.0, 106.0)^c^
CGI-S score	5.0 (5.0, 6.0)^c^

Abbreviations: BMI, body mass index; SSD, Schizophrenia Spectrum Disorders; PANSS, Positive and Negative Syndrome Scale; CGI-S, Clinical Global Impression–Severity scale. ^a^ Data are presented as mean ± SD unless otherwise indicated. ^b^ Age at screening visit. ^c^ Data are presented as median (Q1, Q3).

**Table 2.** **The summary of concomitant medications among patients**.

Medication categories	Number of cases (percentage)
Antipsychotics	121 (53.07%)
Mood stabilizers	28 (12.28%)
Antidepressants	15 (6.58%)
Anxiolytics	7 (3.07%)
Sedative-Hypnotics	50 (21.93%)
Antiextrapyramidal symptom medications	49 (21.49%)
β-Adrenergic antagonists	8 (3.51%)
Glucose-lowering agents	6 (2.63%)
Laxatives	13 (5.70%)

### 3.2 Efficacy Outcomes 

**Fig. 2.**
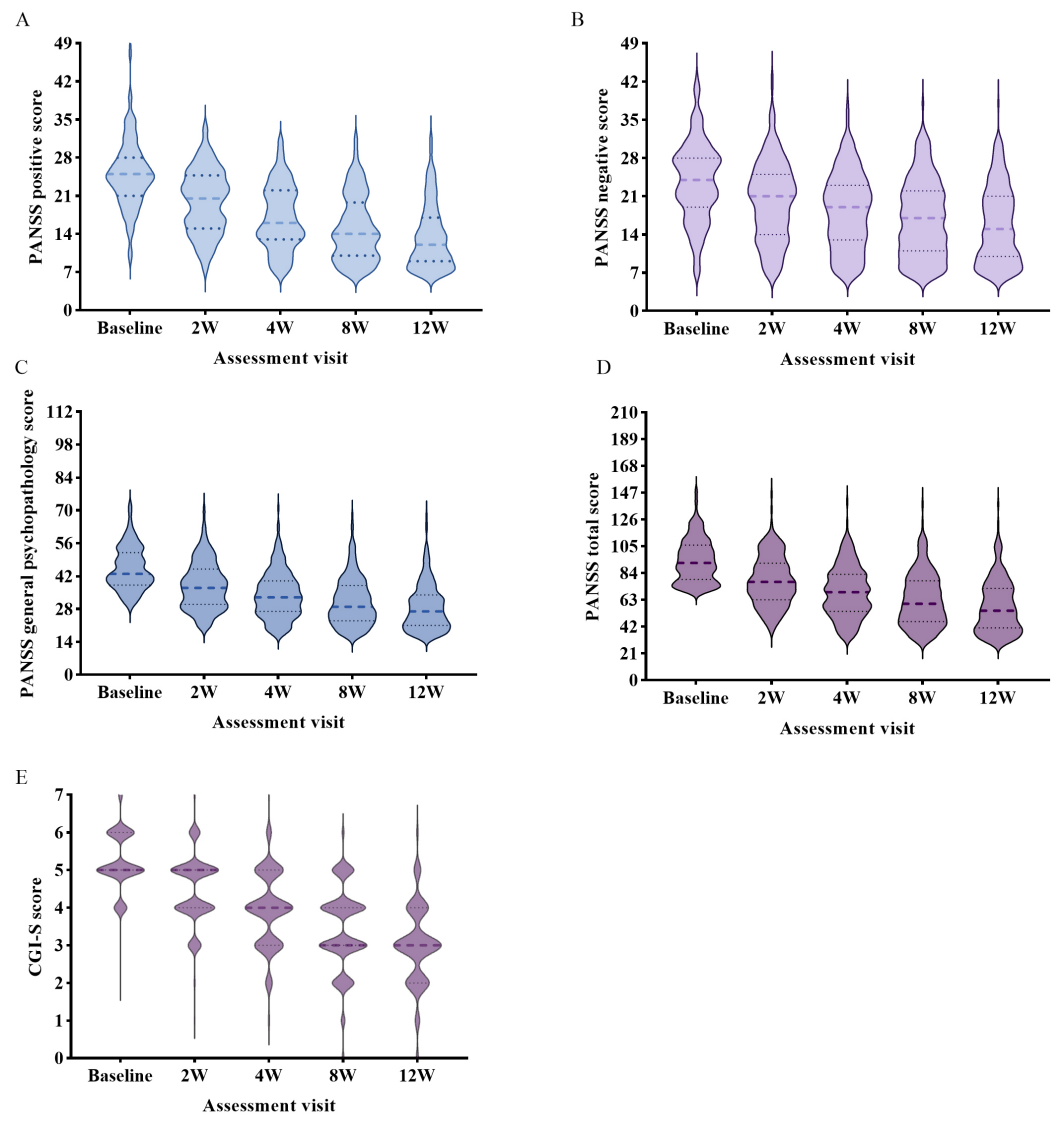
**PANSS and CGI-S scores over time from the baseline to the end of the real-world study**. (A) PANSS positive score in every assessment visit. (B) PANSS negative score in every assessment visit. (C) PANSS general psychopathology score in every assessment visit. (D) PANSS total score in every assessment visit. (E) CGI-S score in every assessment visit. CGI-S, Clinical Global Impression–Severity Scale (score range, 1–7 and baseline range, 1–5, with higher scores indicating more severe illness); PANSS, Positive and Negative Syndrome Scale (score range, 30–210, with higher scores indicating greater symptom severity).

After the treatment with RMI-II, the PANSS total score decreased significantly by 14.02 (3.22, 24.85) at week 2 (*p* < 0.001), indicating effectiveness during the early stage and further decreased by 38.28 (19.02, 51.34) at the endpoint of 12 weeks (*p* < 0.001), demonstrating more significant improvement in various psychotic symptoms. Additionally, positive subscale scores, negative subscale scores, and general psychopathology subscale scores showed statistically significant reductions compared to baseline at all follow-up time points (Fig. 2, Table 3).

**Table 3.** **Difference score values in PANSS Scores over time from Baseline**.

Assessment visit	PANSS scale (n = 228)				
	Total score	Positive score	Negative score	General score	*p* value
Week 2 visit	14.02 (3.22, 24.85)	3.96 (1.09, 7.54)	2.47 (0, 5.27)	6.58 (1.16, 12.26)	<0.001**
Week 4 visit	24.00 (9.55, 36.69)	7.40 (3.13, 11.57)	4.74 (0.91, 8.54)	11.11 (8.52, 16.98)	<0.001**
Week 8 visit	32.75 (13.43, 45.44)	10.38 (4.41, 14.47)	6.25 (1.64, 10.82)	15.10 (6.11, 20.55)	<0.001**
Week 12 visit	38.28 (19.02, 51.34)	12.50 (6.73, 16.19)	7.47 (2.08, 12.05)	17.04 (9.54, 22.65)	<0.001**

Positive score, Negative score, General score indicated the scores of PANSS Positive, negative, and general psychopathology subscales. Difference score values are presented as median (Q1, Q3). ** Compared to baseline, the PANSS total score and subscale score in every assessment visit were significantly decreased after Bonferroni correction (*p* < 0.05/4).

At baseline, over 89% of patients exhibited disease severity distributed in the moderate to severe range, with CGI-S score of 5.0 (5.0, 6.0) points. By week 2 of treatment, the score decreased to 5.0 (4.0, 5.0) points, indicating initiation of symptomatic relief. At the endpoint of week 12 of treatment, the score showed a marked reduction to 3.0 (2.0, 4.0) points, with 46% of patients achieving disease severity below moderate level, demonstrating statistically significant symptomatic improvement compared to baseline (*p* < 0.001).

At weeks 2, 4, 8, and 12 of treatment, the therapeutic response rates (defined as ≥20% reduction in PANSS total scores [23]) were 37.1%, 62.0%, 75.7%, and 83.7% respectively. By week 12 of treatment, 68.9% of subjects achieved clinical remission (defined as scores ≤3 points on PANSS items P1 [Delusions], P2 [Conceptual disorganization], P3 [Hallucinatory behavior], N1 [Blunted affect], N4 [Social withdrawal], N6 [Lack of spontaneity and flow of conversation], G5 [Mannerisms and posturing], and G9 [Unusual thought content] [24]).

### 3.3 Safety Outcomes 

The antipsychotic supplementation dose and duration were dependent on symptom exacerbation and the investigator’s judgment. Permitted oral antipsychotic medications included oral risperidone, oral paliperidone, and others such as olanzapine, amisulpride, aripiprazole, chlorpromazine, etc. (Table 2).

The drug exposure analysis was conducted using the safety analysis set (SS). A total of seven medication administration records were collected during this study. Of the 228 patients in the SS who initiated treatment, 67.84% underwent cross-titration switching, 19.82% utilized direct switching, and 12.33% were treatment-naïve patients. Patients who completed all seven medication administrations represented the majority (62.67%) across medication frequency categories.

Treatment-emergent adverse events (AEs) occurred in 15.35% of patients, and drug-related AEs occurred in 6.58%. Adverse reactions with incidence rates exceeding 1% included extrapyramidal symptoms (EPS) (1.32%) and hyperprolactinemia (1.32%). All reported AEs were of mild to moderate intensity, with no serious adverse events (SAEs) were reported and no patients discontinued treatment due to adverse events (Table 4, Ref. [25, 26]). Weight increased marginally (–0.92 [–2.33, 0.26] kg, *p* = 0.15), with 0.88% of patients experiencing ≥7% weight gain. No significant fluctuations in blood pressure were observed among all participants during the study period, with no statistically significant differences detected between pre-treatment and post-treatment measurements (Table 5).

**Table 4.** **AEs related to RMI-II among patients**.

AEs related to RMI-II		SS (n = 228)	
		Number of cases (percentage)	Number of events
Laboratory/clinical assessments		9 (3.95%)	12
	Prolactin elevation^1^	3 (1.32%)	3
	Weight gain	2 (0.88%)	3
	Leukopenia	1 (0.44%)	1
	Electrocardiogram abnormalities	1 (0.44%)	1
	Tachycardia	1 (0.44%)	1
	Hyperglycemia	1 (0.44%)	1
	Hyperlipidemia	1 (0.44%)	1
	Elevated ALT/AST	1 (0.44%)	1
Neurological disorders		3 (1.32%)	3
	Extrapyramidal disorders	2 (0.88%)	2
	Akathisia	1 (0.44%)	1
Endocrine disorders		2 (0.88%)	2
	Hyperprolactinemia^2^	2 (0.88%)	2
Cardiac disorders		2 (0.88%)	2
	Bifascicular block	1 (0.44%)	1
	Palpitations	1 (0.44%)	1
Gastrointestinal disorders		1 (0.44%)	1
	Constipation	1 (0.44%)	1
Hematologic and lymphatic disorders		1 (0.44%)	1
	Anemia	1 (0.44%)	1

Abbreviations: AEs, adverse events; RMI-II, Risperidone Microspheres for Injection (II); SS, Safety set; ALT, Alanine Aminotransferase; AST, Aspartate Aminotransferase. The rates of AEs were based on the sample (SS). ^1^Prolactin elevation: prolactin levels exceeding 25 ng/mL without associated clinical symptoms [25, 26]. ^2^Hyperprolactinemia: prolactin levels exceeding 25 ng/mL with reproductive dysfunction, sexual impairment, or breast pathology [25, 26].

**Table 5.** **Blood pressure at each assessment visit**.

Assessment visit	Systolic blood pressure (mmHg)	*p* value	Diastolic blood pressure (mmHg)	*p* value
Baseline	120.0 (110.0, 125.0)	/	76.0 (71.0, 80.0)	/
Week 4 visit	120.0 (110.0, 125.0)	0.936	75.0 (70.0, 80.0)	0.419
Week 8 visit	121.0 (112.0, 126.0)	0.560	75.0 (70.0, 81.0)	0.852
Week 12 visit	121.0 (110.0, 127.0)	0.255	76.0 (70.0, 80.0)	0.999

Difference score values are presented as median (Q1, Q3). The blood pressure at each assessment visit was not significantly different from the baseline after the Bonferroni correction (*p* > 0.05/4).

## 4. Discussion

The present real-world study provided robust evidence supporting the clinical utility of RMI-II in managing SSD, demonstrating significant symptom alleviation and favorable tolerance over a 12-week observation period. Our findings aligned with established clinical trial data while offering unique insights into routine practice patterns.

In the acute phase of SSD, more than 20% of patients frequently exhibit psychomotor agitation, prompting effective symptom control during the early stage as a critical determinant of the entire treatment course [27]. The efficacy and safety profile of risperidone in treating acute schizophrenia have been well-established through robust clinical validation [28, 29, 30]. LAIs demonstrate superior therapeutic advantages over oral formulations, including more reliable bioavailability and enhanced patient adherence. Clinical evidence indicates that early initiation of LAI therapy significantly reduces hospitalization rates and treatment discontinuation rates [31]. However, the delayed release kinetics inherent to conventional depot formulations, characterized by a post-administration lag phase in drug release, have historically limited their utility in acute-phase management [32, 33]. In this study, the early-onset efficacy of RMI-II observed at week 2 challenges conventional expectations about delayed therapeutic effects of LAIs. By week 12, the cumulative PANSS reduction exceeded typical randomized controlled trial outcomes [34], potentially reflecting real-world advantages of assured medication adherence through depot administration in chronic populations [35]. Notably, the temporal progression of response rates (37.1% at week 2 *vs.* 83.7% at week 12) mirrored the pharmacokinetic profile of RMI-II, which started improving multidimensional psychiatric symptoms in acute phase SSD, and maintained the good therapeutic effect of long-acting injections of risperidone microspheres [36]. This dose-response correlation reinforced the importance of sustained treatment continuity, particularly given that delayed responders accounted for 46.6% of total responders between weeks 2–12. Such findings emphasize the clinical imperative to maintain therapy beyond initial evaluation windows.

The CGI-S improvement from moderate-severe to mild-moderate severity aligned with functional recovery patterns observed in pragmatic studies [37]. This transition corresponds to clinically meaningful milestones, including regained self-care capacity and reduced caregiver burden [38], though future studies should incorporate functional outcome measures to confirm this association. Previous studies reported LAI drugs were superior to oral antipsychotic drugs in terms of long-term efficacy and social function differences in the treatment of newly diagnosed schizophrenia patients [39, 40], consistent with this study.

In terms of safety, the AEs observed in this study were predominantly EPS and hyperprolactinemia with severity of mild to moderate, and no participant discontinuation due to adverse reactions, indicating favorable tolerance of RMI-II, which is inherently associated with the pharmacological profile of second-generation antipsychotic drugs. These findings are consistent with previous research outcomes from a study investigating earlier formulations of injectable risperidone microspheres [41]. The prolactin-elevating effect of antipsychotic drugs was mediated by their dopamine D2 receptor antagonism in the tuberoinfundibular pathway, with risperidone demonstrating a more pronounced hyperprolactinemic effect compared to other atypical antipsychotics [42]. The present study found that 0.88% patients exhibited increased BMI at week 12, and other metabolic parameters showed no statistically significant differences compared to baseline, suggesting a lower metabolic risk associated with the use of RMI-II in the treatment of SSD. The low incidence of metabolic side effects suggests preserved metabolic advantages compared to other second generation antipsychotics (SGAs) [43], though longer-term monitoring remains essential.

However, there were several limitations in this study. Firstly, the study lacked a control group. Secondly, considering the nature of real-world studies, there was a potential for confounding factors to influence the results. On the one hand, confounding by factors such as age, distance, culture or economy, etc. affected treatment persistence, therefore withdrawn of 79 patients from the study may introduce attrition bias, and we utilized LOCF according ITT analysis principle to compute the missing in the FAS to prevent exaggerating the therapeutic effect. On the other hand, in the real-world study, considering the past medication history and disease characteristics of patients, concomitant medications were allowed, which may cause masking effect bias or AE report bias. Thirdly, sample size constraints may limit detection of rare adverse events or subgroup differences, while external validity could be compromised by site-specific practices or socioeconomic disparities. Fourthly, no cost-effectiveness analysis of the treatment regimen was conducted. Given the relatively high market price of the RMI-II, this may limit the direct applicability of our findings for guiding the optimization of healthcare resource allocation, and treatment costs could impact patient accessibility and influence clinical prescribing decisions. Which is similarly reflected in the current situation of LAIs in China. The rate of use of LAIs to treat patients with schizophrenia in China is significantly lower than the average of other Asian countries/regions due to factors such as the availability of drugs, pharmaco-economics, and prescribing habits of psychiatrists. In the future, policymakers should pay more attention on coordinating the actions of all relevant departments [44]. Additional challenges included potential underreporting of long-term safety risks and long-term efficacy due to the relatively short study period of 12-week, biases from unblinded treatment decisions. However, despite these limitations, few studies have followed patients initiating RMI-II treatment for such a long period in real world. The findings of this study may contribute to provide preliminary evidence on the efficacy and safety profile of RMI-II in SSD population.

## 5. Conclusions

In this study, RMI-II appeared to be effective and well-tolerated. These findings suggest it may represent an additional therapeutic option for SSD. However, further comparative studies are needed to better define its role among available LAIs in clinical practice.

## Availability of Data and Materials

The data sets generated and analyzed during the current study are not publicly available due to privacy and ethical restrictions involving participant data, but are available from the corresponding author on reasonable request. All data access requests will be evaluated in accordance with institutional and ethical guidelines to ensure participant confidentiality.
